# Honeybees show adaptive reactions to ethanol exposure

**DOI:** 10.1038/s41598-018-27117-6

**Published:** 2018-06-07

**Authors:** Krzysztof Miler, Karolina Kuszewska, Valeriya Privalova, Michal Woyciechowski

**Affiliations:** Institute of Environmental Sciences, Jagiellonian University in Kraków, Krakow, Poland

## Abstract

The honeybee is being developed as a simple invertebrate model for alcohol-related studies. To date, several effects of ethanol consumption have been demonstrated in honeybees, but the tolerance effect, one of the hallmarks of alcohol overuse, has never been shown. Here, we confirm our hypothesis that the response to ethanol (in terms of motor impairment) is lower in bees that have previously experienced intoxication than in bees encountering ethanol for the first time, indicating that the chronic tolerance effect occurs in honeybees. Furthermore, we investigated the basis of this effect and found that it likely results from conditioned compensatory responses to cues associated with ethanol delivery. Our findings significantly improve our understanding of the suitability of honeybees as models for alcoholism-related research and underline the first and foremost function of all conditioned reactions – their adaptive value.

## Introduction

Alcoholism research can rapidly advance if reliable invertebrate models are available for the study of alcohol-related behaviours and predispositions^1,2^. The honeybee *Apis mellifera* is being developed as a simple invertebrate model for alcohol-related studies. Its elaborate behavioural repertoire, excellent learning skills, advanced sociality, possession of ethanol-degrading enzymes, and well-known neuroanatomy, as well as the fact that it encounters ethanol in nature, make it a potentially valuable source of insight^1,2^. To date, ethanol has been found to have several effects on honeybees in both social and non-social behaviours^3–17^. Honeybees have been demonstrated to readily consume ethanol both in captivity and in the field^3,5,13,16^. Ethanol consumption results in impaired locomotion, learning, communication and foraging decisions^3,7,8,10,12–15^, as well as increased aggression, analgesia and self-grooming^6,9,10,12,17^. In addition, ethanol consumption tendencies and the effects of ethanol intake have been shown to be at least partly blocked by appropriate drugs^4^ and to reflect ethanol levels in the haemolymph^11,13^. Honeybees have even been demonstrated to feed each other ethanol retained in the crop and spread intoxication within the hive^17^. In light of these results, it is clear that honeybees show promise as a model for alcohol-related behavioural investigations.

In the present study, we investigated whether honeybees develop tolerance to ethanol. Ethanol tolerance is a hallmark of alcohol abuse^1,2^, and this effect has never been shown in bees. There are two mechanisms of tolerance (which are not mutually exclusive): *metabolic* (less efficient absorption and/or more efficient removal of ethanol) and *functional* (adaptation in neural functions to ethanol exposure)^18^. Our study was not aimed at determining how these mechanisms function in ethanol-intoxicated honeybees but rather at measuring the potential changes in the motor performance of these insects after being repeatedly exposed to ethanol, i.e., determining whether honeybees show *chronic* tolerance to ethanol. Our results indicate that tolerance, displayed as an increase in resistance to the impairing influence of ethanol on motor performance, occurs in honeybees. Furthermore, our findings suggest that conditioned compensatory responses are likely responsible for this effect by countering the impairing effects of ethanol intoxication, thus lowering the sensitivity to the drug.

## Methods

**Figure 1:**
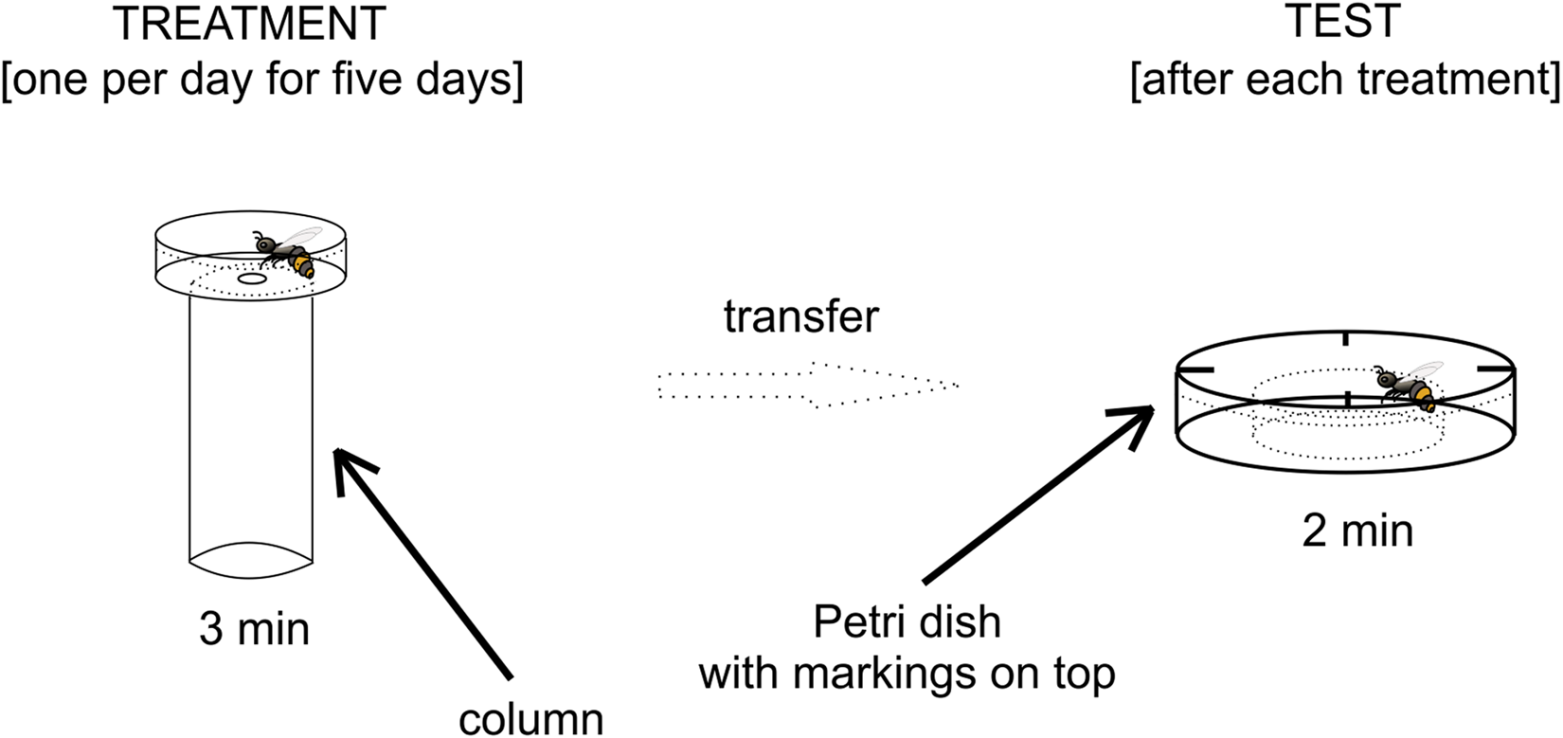
The schema of the first experiment. Treatment was conducted on four groups of bees, individually, once per day for five consecutive days. Depending on the group and the day, there was either 30 ml of water or ethanol in the treatment column. After treatment, each individual bee was transferred into the test Petri dish with markings on top. There, its motor performance was assessed for two minutes (i.e., the number of markings crossed).

In the first experiment, we used four honeybee colonies. The experiment was conducted one colony at a time (four weeks during May and June 2017). Newly emerged bees from a given colony were divided into four groups (40 individuals per group). These groups were held in separate experimental cages and placed in an incubator at 32 °C for 72 h without any treatment, with water and sucrose solution available ad libitum. After this acclimatization period, the treatments started and lasted for five days (one treatment per day). For treatment, a given experimental cage was taken out of the incubator, and a single bee was individually closed inside a transparent Petri dish (3.5 cm in diameter) with a hole (0.5 cm in diameter) in the bottom. The dish was then placed on a column (3.0 cm in diameter) filled with 30 ml of room-temperature water or 98% ethanol (depending on the group). After three minutes, the bee was taken out of the Petri dish and transferred to a test arena. The test arena was made of two Petri dishes, one larger (5.5 cm in diameter) and one smaller (3.5 cm in diameter), with the smaller dish placed in the centre of the larger one to create a corridor between their walls (divided into quarters by markings on top of the larger dish). Each 2-minute test started after a bee was placed inside this corridor. During the test, we measured the number of markings crossed. After testing, the bees were returned to the incubator in their experimental cages until the procedure was repeated the next day. The procedure was conducted each time in a laboratory setting between 8 AM and 8 PM at a constant 23 °C temperature and 50–70% relative humidity. Over the course of the experiment, individual bees showing impaired movement for reasons unrelated to the experimental treatment (e.g., a missing limb) were excluded. See Fig. 1 for the experimental setup schema.

As mentioned, four groups were established in each colony. The treatment column contained water for five days in group one (G1), water for four days and then ethanol on the fifth day in group two (G2), ethanol for five days in group three (G3), and ethanol for four days and then water on the fifth day in group four (G4). These conditions enabled us to investigate the behaviour of bees that were exposed to water vapour (G1), exposed to ethanol vapour without prior experience with this substance (G2), repeatedly exposed to ethanol vapour (G3), or exposed to water vapour after prior experience with ethanol (G4). Only individuals who lived until the final test were included in the final dataset for all days and groups (e.g., an individual that died on day four was excluded from the final dataset). We were able to track the bees individually because we marked each one with a number. Thus, we avoided any potential bias from including weak, short-lived individuals in our analyses.

We used a generalized linear mixed model (GLMM) to analyse our results (dependent variable: number of markings crossed [motor performance]; fixed factors: group, day, and group x day; random factors: colony and individuals nested within colony). To test the significance of the group x day interaction, we applied a post hoc HSD test for unequal numbers. The statistical analysis was performed with the SPSS programme (IBM, Poland).

**Figure 2:**
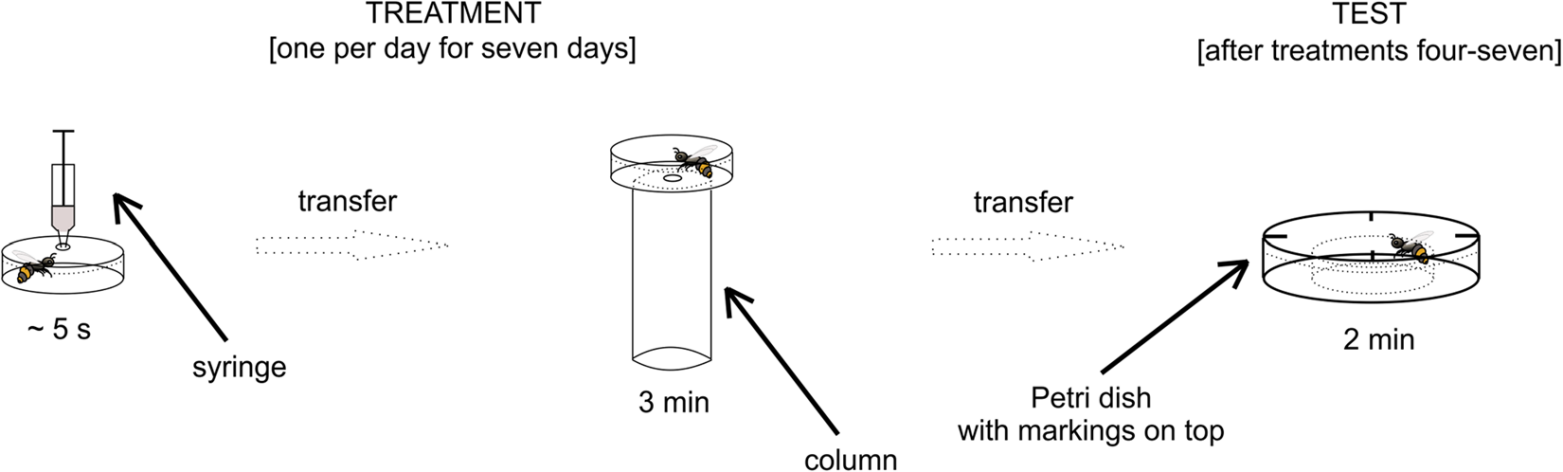
The schema of the second experiment. Treatment was conducted on five groups of bees, individually, once per day for seven consecutive days. Depending on the group and the day, one of two odours was delivered by pressing the syringe with a scented cotton plug inside, followed by exposure to water or ethanol on the treatment column (30 ml of either water or ethanol in the column). After treatment, on days four to seven, each individual bee was transferred into the test Petri dish with markings on top. There, its motor performance was assessed for two minutes (i.e., the number of markings crossed).

In the second experiment, we used another four honeybee colonies (previously unused; the experiment was conducted one colony at a time for four weeks in June and July 2017). Bees were prepared as in the first experiment, but five groups were created in each colony (50 individuals per group). The treatments started after the acclimatization period and lasted for seven days (one treatment per day). For treatment, a given experimental cage was taken out of the incubator, and a single bee was closed inside a transparent Petri dish (3.5 cm in diameter), given a 3-ml odour puff and left in place for ~5 seconds. Odour was delivered through a small hole in the top of the dish, and a syringe containing a cotton plug inside with a drop of either 2-octanone or 1-hexanol (depending on the day) was attached to the hole. The plunger of the syringe was pressed steadily for odour delivery. Pure substances used as odourants were obtained from Sigma-Aldrich (USA). The bee was then transferred to an identical Petri dish with a hole (0.5 cm in diameter) in the bottom and placed on a column (3.0 cm in diameter) filled with 30 ml of room-temperature water or 98% ethanol (depending on the group). After 3 minutes, the bee was removed from the Petri dish and transferred to a test arena. The test arenas were identical to the ones used in the first experiment, and the tests were performed in the same manner. Again, as in the first experiment, the tested bees were returned to the incubator in their experimental cages until the procedure was repeated the next day. The procedure was conducted each time in laboratory conditions identical to the ones in the first experiment, again between 8 AM and 8 PM. Similarly, any individual showing impaired movement for reasons unrelated to the experimental treatment (e.g., a missing limb) was excluded. See Fig. 2 for the experimental setup schema.

Five groups were established in each colony (see Table 1). In this experiment, we investigated the behaviour of bees that were exposed to water vapour under fixed odour conditions (G1), to water vapour under changed odour conditions (G2), to ethanol vapour under fixed odour conditions (G3), to water vapour in odour conditions previously associated with ethanol vapour exposure (G4), or to water vapour in odour conditions differing from those associated with ethanol vapour exposure (G5). In two out of four colonies, we conditioned bees to 2-octanone as the fixed odour (with 1-hexanol as the changed odour), and in the other two, we used 1-hexanol as the fixed odour (with 2-octanone as the changed odour) (see Table 1). As in the first experiment, only individuals who lived through the final test were included in the final dataset for all days and groups (the bees were individually marked). In this experiment, we measured performance (number of markings crossed) only on days four through seven. The statistical analysis was identical to the one performed for the first experiment.

**Table 1:** Experimental design of the second experiment.

	Day 1	Day 2	Day 3	Day 4	Day 5	Day 6	Day 7
G1	H_2_O fixed	H_2_O fixed	H_2_O fixed	H_2_O fixed	H_2_O fixed	H_2_O fixed	H_2_O fixed
G2	H_2_O fixed	H_2_O fixed	H_2_O fixed	H_2_O fixed	H_2_O changed	H_2_O changed	H_2_O changed
G3	EtOH fixed	EtOH fixed	EtOH fixed	EtOH fixed	EtOH fixed	EtOH fixed	EtOH fixed
G4	EtOH fixed	EtOH fixed	EtOH fixed	EtOH fixed	H_2_O fixed	H_2_O fixed	H_2_O fixed
G5	EtOH fixed	EtOH fixed	EtOH fixed	EtOH fixed	H_2_O changed	H_2_O changed	H_2_O changed

The experiment lasted seven days and included five groups, with a single treatment per day in each group. “H_2_O” and “EtOH” denote water and ethanol, respectively, in the treatment column. Below, information is given on the type of odour delivered prior to the treatment on the column.

### Data availability statement

Relevant data are within the paper Supplementary Datasets.

## Results and Discussion

**Figure 3:**
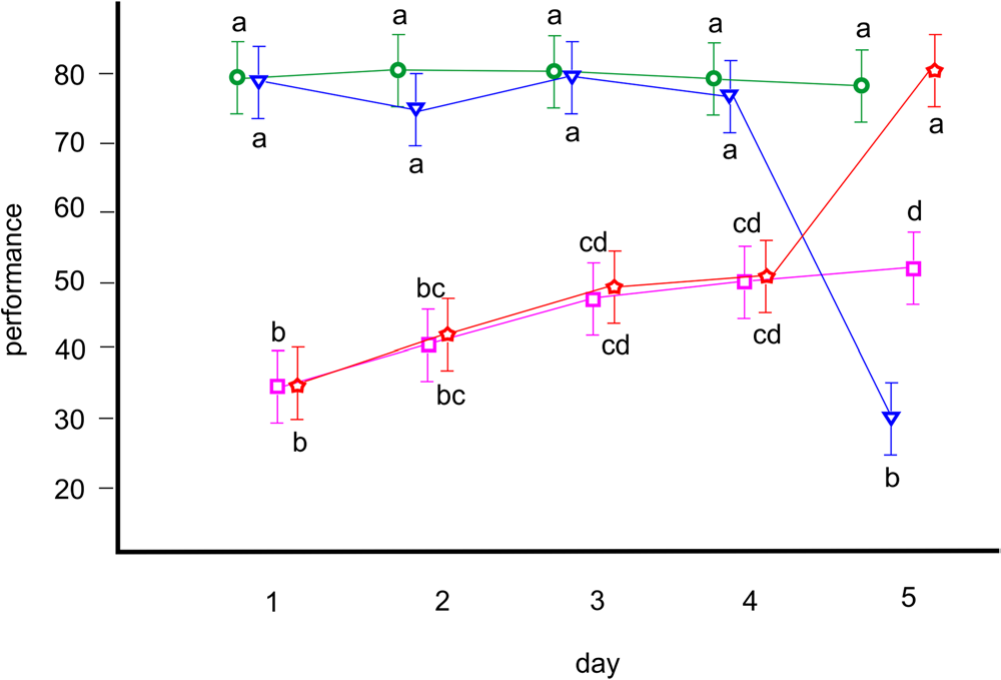
The results of the first experiment. Motor performance of individual bees in group one (green circles; exposed to water vapour every day), group two (blue triangles; exposed to water vapour on days one to four and then to ethanol vapour on day five), group three (magenta squares; exposed to ethanol vapour every day) and group four (red stars; exposed to ethanol vapour on days one to four and then to water vapour on day five). Lower performance indicates impaired movement. Shapes (circles, triangles, squares and stars) denote mean values, and whiskers denote 95% confidence intervals. Letters denote the results of the post hoc comparisons (different letters show significant differences). Group one (green circles) did not show any significant differences between days, whereas group two (blue triangles) showed a significant drop in performance when exposed to ethanol for the first time on the last day of the experiment. Group three (magenta squares) showed low performance with steady significant improvement over time, similar to group four (red stars), which showed the same low performance with steady and significant improvement until it was exposed to water instead of ethanol on the last day of the experiment. At that time, group four displayed a marked increase, matching its performance to group one (green circles).

**Figure 4:**
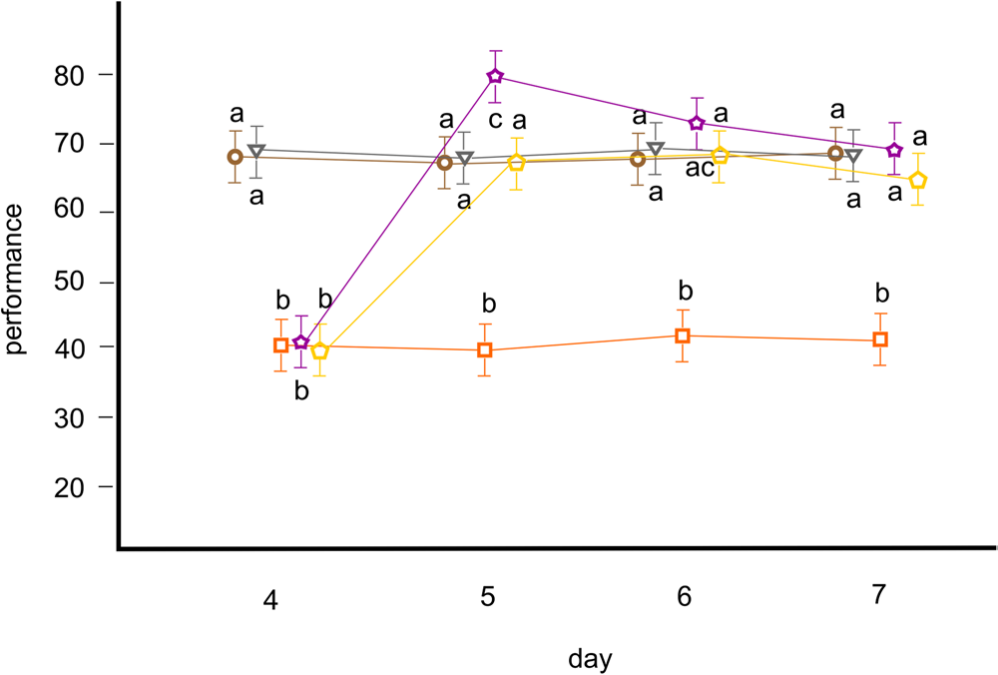
The results of the second experiment. Motor performance of individual bees in group one (brown circles; exposed to water vapour on days one to seven, always with the same odour), group two (grey triangles; exposed to water vapour on days one to seven, with the odour changed on day five), group three (orange squares; exposed to ethanol vapour on days one to seven, always with the same odour), group four (purple stars; exposed to ethanol vapour on days one to four and to water vapour on days five to seven, always with the same odour) and group five (yellow pentagons; exposed to ethanol vapour on days one to four and to water vapour on days five to seven, with the odour changed on day five). Lower performance indicates impaired movement. Shapes (circles, triangles, squares, stars and pentagons) denote mean values, and whiskers denote 95% confidence intervals. Letters denote the results of the post hoc comparisons (different letters show significant differences). Group one (brown circles) did not show any significant differences between days, similarly as group two (grey triangles). Group three (orange squares) showed steady low performance over time. Group four (purple stars) showed high improvement when, on day five, it was exposed to water instead of ethanol, in fixed odour conditions, and outperformed both control groups one and two (brown circles and grey triangles, respectively). Improvement matching the performance of the control groups was observed on day five in group five (yellow pentagons) when exposed to water instead of ethanol and when odour conditions were changed. The difference between group four (purple stars) and groups one, two and five (brown circles, grey triangles and yellow pentagons, respectively) observed on day five disappeared completely by day seven.

In the first experiment, a GLMM for motor performance, with the group and day as fixed factors, showed that both were significant (F_1,3_ = 134.518, p < 0.001 and F_1,4_ = 45.813, p < 0.001, respectively), as was their interaction (F_1,12_ = 45.439, p < 0.001; see Fig. 3). Furthermore, in the second experiment, another GLMM for motor performance, with the group and day as fixed factors, showed that both were significant (F_1,4_ = 120.851, p < 0.001 and F_1,3_ = 95.968, p < 0.001, respectively), as was their interaction (F_1,12_ = 38.851, p < 0.001); see Fig. 4.

The results of the first experiment indicated that bees exposed to ethanol vapour showed impaired movement, but this effect was strongest upon their first encounter with the drug. There was a clear increase in the motor performance of bees repeatedly exposed to ethanol vapour, although the performance in the third group appeared to reach a plateau (Fig. 3; magenta squares), indicating that most of the individuals had reached their tolerance capacity. This chronic tolerance may well have been learned, i.e., resulting from Pavlovian conditioning to cues associated with ethanol^19–21^. In such a case it would appear that ethanol impairs the motor performance of bees (e.g., by the decrease in coordination), which serves as the unconditioned stimulus to which bees respond unconditionally by increasing their motor performance (e.g., by improving coordination). In the second experiment, we detected such increase in motor performance by creating conditions for additional conditioning. Bees showed motor overperformance when experiencing ethanol-associated odour in ethanol-free conditions (Fig. 4; group four, marked with purple stars on day five) but did not show such an effect in ethanol-free conditions when they experienced a changed odour that was not associated with ethanol exposure (Fig. 4; group five, marked with yellow pentagons on day five). This conditioned compensation, together with an unconditional increase in motor performance, likely serves as the basis of the chronic tolerance effect reported here. This result has been demonstrated previously, e.g., in rats, which develop similar reactions to morphine. Rats appear to show decreasing analgesic effects upon repeated treatment with morphine (a chronic tolerance effect) but become conditioned to the morphine injections and thus show hyperalgesia when they receive a saline injection instead of a morphine injection^22^. Clearly, such effects are observed because drugs, such as morphine and ethanol, disturb the homeostasis of the organisms they act upon. Both rats and bees develop responses that lower the disturbing effect of the drug, which is seen as tolerance development. Overall, these results are in accordance with the classical conditioning model of tolerance proposed for humans^23^ and are in line with results obtained from both humans and rats^24–29^.

Note that we observed compensation only in the second experiment. In the first experiment, we may have detected some compensatory reactions in group four, but overall, that group performed similarly to the control on the last day (Fig. 3; red star and green circle, respectively, on day five). However, there was a high variance in performance within group four, with some bees performing very well on the last day. Additionally, note that compensation diminished rapidly (Fig. 4; purple stars on days five to seven). Extinction, in this context, is what one would expect if the effect was conditioned^30^, as we suggest.

Our data indicate that honeybees develop ethanol tolerance similar to *Drosophila* and *Caenorhabditis*, two invertebrate models used in alcohol research^31–33^. In this aspect, these organisms behave similarly to mammals^34^. Importantly, however, we are yet unable to determine the relative contribution of metabolic and functional mechanisms in the chronic tolerance to ethanol reported here^18^.

Our findings expand the range of knowledge regarding honeybees as models in alcohol research^1,2^. Similarly to other pollinators^35–37^, honeybees encounter ethanol in nature, in fermenting nectar, honey and fruits, although they digest it orally and in low percentages. Here, we chose vapour as the mode of delivery of ethanol, using a setup similar to that used by Ammons and Hunt^9^ to investigate honeybee sensitivity to ethanol and its correlation with aggression. As these authors note, intoxicating bees by exposing them to ethanol vapour presents a great advantage of bypassing the side effects of ethanol digestion or metabolism. Furthermore, ethanol vapour was used in studies investigating *Drosophila* flies and their reactions to ethanol^38^, proving extremely useful. Thus, our experimental setup may be viewed as an advantage rather than a disadvantage.

Overall, we have shown that honeybees exhibit a chronic tolerance effect to ethanol, expressed in terms of motor performance. Furthermore, we suggest that Pavlovian conditioned compensation is a probable and important component of the mechanism of this tolerance. Clearly, our study exposes gaps in knowledge and a need for further studies on alternate forms of tolerance displayed by honeybees, including assessing metrics other than motor performance. Furthermore, our results open interesting research avenues, such as the investigation of the molecular and neuroanatomical bases of the functional component of reported tolerance.

## Electronic supplementary material

Dataset 1 with the results of the first experiment 

Dataset 2 with the results of the second experiment
